# Professor H. B. Sands, M. D.

**Published:** 1888-12

**Authors:** 


PROFESSOR H. B. SANDS, M. D.—In the death of Dr. H. B. Sands, at the comparatively early age of fifty-eight, New York loses her most prominent surgeon. As a teacher, his lectures were clear and forcible, and occasionally spiced with genial and clean humor. He was justly popular with his classes. He had a large professional experience, his judgment was excellent, and he was a good and, what is more, a conscientious operator. His acquirements covered all that was within easy reach. He used well what others had gained and what was well known and certain, without worrying about the unknown or doubtful, or contributing much himself. An able, safe, and conservative practitioner, he reaped the reward of his wise expenditure of strength in gaining a high social position, many warm friends, and a large and lucrative practice. L. C.

